# Human-robot planar co-manipulation of extended objects: data-driven models and control from human-human dyads

**DOI:** 10.3389/fnbot.2024.1291694

**Published:** 2024-02-12

**Authors:** Erich Mielke, Eric Townsend, David Wingate, John L. Salmon, Marc D. Killpack

**Affiliations:** Robotics and Dynamics Laboratory, Brigham Young University, Mechanical Engineering, Provo, UT, United States

**Keywords:** physical human-robot interaction, force control, cooperative manipulation, learning and adaptive systems, human-robot interaction, neural network, variable impedance

## Abstract

Human teams are able to easily perform collaborative manipulation tasks. However, simultaneously manipulating a large extended object for a robot and human is a difficult task due to the inherent ambiguity in the desired motion. Our approach in this paper is to leverage data from human-human dyad experiments to determine motion intent for a physical human-robot co-manipulation task. We do this by showing that the human-human dyad data exhibits distinct torque triggers for a lateral movement. As an alternative intent estimation method, we also develop a deep neural network based on motion data from human-human trials to predict future trajectories based on past object motion. We then show how force and motion data can be used to determine robot control in a human-robot dyad. Finally, we compare human-human dyad performance to the performance of two controllers that we developed for human-robot co-manipulation. We evaluate these controllers in three-degree-of-freedom planar motion where determining if the task involves rotation or translation is ambiguous.

## 1 Introduction

In the future, robots will work alongside humans in many applications including logistics, health-care, agriculture, disaster response, and search and rescue. The advantage of human-robot collaboration in these areas is that humans provide intelligence and dexterity while robots may provide strength, stability, and even redundancy (Kazerooni, 1990). Physical Human-Robot Interaction (pHRI) for collaborative manipulation (or co-manipulation) is an area of robotics that can especially benefit from the combined strengths of a human-robot team: strength and execution from the robot and intelligence and planning from the human. This is particularly true of co-manipulation tasks where a human and a robot physically manipulate the same object simultaneously. Co-manipulation can include complex translational and rotational tasks, such as moving a table (Mörtl et al., 2012), couch, or other extended, rigid object. These objects may be heavy or unwieldy, which could necessitate two or more people to carry them. A robot capable of replacing a human in these teams would help in situations like search and rescue where current high-payload robots are too heavy and dangerous to relocate and operate. Robots that can physically interact with a human could help lift and remove rubble from disaster areas or take a victim on a stretcher to safety. These robots would allow fewer people to complete the same amount of work, or for more teams to operate and reach more people in need of help. Other applications include using robots to help load and unload moving vans, using robots to help move objects around warehouses, and any other co-manipulation applications where human-human teams are currently needed.

In these situations, robots will need to work safely and intuitively, in order to be an asset when interacting with people. However, often a task is poorly defined for one or both partners of a dyad. Uncertainty or ambiguity can especially exist when tasks include manipulating an extended object that may need to be either translated or rotated, or both. In order to be effective, a pHRI robot controller for co-manipulation of extended objects must be able to distinguish between an intent to rotate and translate. By studying human-human interaction (HHI) data, we can define patterns that will help to create a safe and intuitive co-manipulation controller. Therefore, this paper proposes a method for predicting human intent in a co-manipulation task based on HHI. We designate human intent as the intent to move an object in a particular direction along a trajectory with a particular velocity.

There are many signals that could be used to predict human intent, including motion, force, partner posture, and verbal communication among others. In our study, we chose to focus on motion and force, since we expect that these variables are the most fundamental and easiest for a robot to sense and interpret. This does not mean that other information sources could not be used to improve upon our results, but rather that this data is sufficient to characterize human intent in co-manipulation tasks. Further, other studies have confirmed that haptic-channel communication is sufficient to indicate motion intent (Sawers et al., 2017). However, while the past work on co-manipulation outlined in Section 2 shows that collaboration through force is applicable to some tasks, it is not clear that previously developed algorithms and intent-estimators will work in cases that include whole-body, bi-manual, six degree of freedom (DoF) manipulation of an object, rather than planar arm movements only. The initial goal of our co-manipulation controllers is to know how the robot should move, based on sensory inputs, in order to manipulate the object being carried in the manner desired by the human partner. By basing our human-intent model on data from human-human dyads, we are increasing the likelihood that our controller will be intuitive for human users.

The specific contributions of this paper include the following:

1. Observations on planar motion from a human-human co-manipulation study (see Section 3.2), which include the following:
  - Lateral movements are triggered by a specific torque sequence.
  - Planar rotation movements can be distinguished from lateral movements using sequences of applied torque.
2. Development of a neural network to predict human intent based on past motion (see Section 4.3).
3. Application of the neural network and trigger-based predictions to a human-robot dyad, comparing the performance of human-robot dyads with human-human dyads (see Section 5).

We next outline the organization of the rest of this paper. Section 2 describes related work on physical human-robot interaction and intent modeling. Next, the human-human dyad experiment is explained in Section 3, including the main results and observations of the HHI study. Section 4.1 describes the robot hardware used in our co-manipulation experiments. In Section 4.2 we discuss the formulation and preliminary testing of an Extended Variable-Impedance Controller for human-robot co-manipulation. We then describe the structure, training, and validation of a neural network, as well as the formulation of a neural-network-based controller in Section 4.3. In Section 4.4 we describe a physical human-robot co-manipulation experimental study comparing both human-robot controllers. Finally, we discuss the results of the human-robot study in Section 5 with conclusions in Section 6.

## 2 Related work

In this literature review, we group the efforts of past researchers into a few different categories: studies about co-manipulation or human behaviors, force-based and motion-based co-manipulation methods, determining the performance of human-robot dyads through metrics, and human intent estimation.

### 2.1 Co-manipulation and human behavior studies

One of the most widely used studies that explore human-arm reaching movement was performed by Flash and Hogan (1985). They illustrated the tendency of upper-arm reaching movements to resemble minimum-jerk trajectories. Another fundamental study was performed by Rahman et al. (1999) where they performed a 1 DoF translation co-manipulation experiment between two human users, showing that users exhibited variable impedance along with minimum-jerk trajectories.

There were also a number of studies investigating how humans cooperate through forces and haptic channels. In particular, Reed et al. (2007); Wel et al. (2011), and Ganesh et al. (2014) all showed that human-human dyads were able to perform better than when performing the task as individuals. However, when Reed et al. included a robot, this advantage disappeared.

Focusing on trying to understand conflicts in human-human interaction to better enable future human-robot interaction, in Madan et al. (2015) the authors use haptic devices and a virtual collaborative task to explore haptic interaction patterns related to quantifying consensus in a dyadic interaction. While in Al-Saadi et al. (2020) they used wrench-based metrics and divided interaction patterns into discrete behavior classes describing how a dyad was working for translational and rotational tasks. A major difference in our paper, where we move a large, heavy object which requires bi-manual manipulation, is that we use haptic or motion signals to generate robot motion commands directly, with the sole objective of making the robot an effective follower.

Other studies have shown that a haptic channel can be used as the only source of information exchange between partners. Sawers et al. (2017) performed an experiment where participants performed a series of dance steps with a partner while Mojtahedi et al. (2017) showed that interaction forces may communicate movement goals between human-human dyads in cooperative physical interactions.

One of the only studies performed with a human-human dyad carrying an extended object was done by Bussy et al. (2012b). In this experiment, they had dyads move a beam in 1 DoF, both forward and backward and used object velocity to trigger state transitions in a state machine model.

### 2.2 Control methods for co-manipulation

#### 2.2.1 Force-based co-manipulation methods

One of the first controllers for cooperative manipulation of an object by robots and humans was an impedance controller developed by Ikeura et al. (2002) and Rahman et al. (2002). They also developed strategies for situations that required using direction of force and change in magnitude of force. This type of control technique is known as variable-impedance control (Ikeura and Inooka, 1995; Dimeas and Aspragathos, 2015). The defining characteristic of this method is measuring Cartesian-coordinate forces at the end effector to determine motion intent in certain Cartesian directions. Tsumugiwa et al. (2002) showed that varying the impedance allows for increased performance of human-robot interaction in calligraphy. This variable impedance approach was also very successful in predicting Cartesian movements, as was shown in other studies as well (see Duchaine and Gosselin's, 2007; Ficuciello et al., 2015). However, it does not generalize to include rotational movements. It also is heavily dependent on human force input, meaning the robot does not proactively contribute to moving the object being manipulated, and the sole human partner must exert more force than may be required in a human-human dyad.

The initial work in variable impedance control (VIC), however, provided a basis for using haptic information in future pHRI controllers. One such controller was implemented by Ranatunga et al. (2016) who performed 1 DoF point-to-point motion tasks without previous knowledge of the trajectory, which is necessary for situations such as search and rescue. However, the work assumed direct contact between human and robot, (i.e. no extended object co-manipulation), and was limited in DoF. Further, there is an inherent problem with VIC, and other methods, such as Leica et al. (2013)'s method for moving extended objects, that limits how many DoFs are viable. This is known as the translation versus rotation (TvR) problem. In a simple planar task, the leader has the option of moving the extended object by either translating forward/backward, translating laterally, or rotating the object. The problem arises when the leader wishes to move laterally, and so applies a force in that direction. The follower, who is positioned some distance away from the applied force, perceives the force as a torque, and begins to rotate the board. This shows that there is information missing in VIC to deal with the TvR problem.

Two approaches to solve this problem were suggested by Karayiannidis et al. (2014) and Nguyen (2016). Karayiannidis et al. used the direction and magnitude of the applied force to an extended object to create a state machine that switches between translation and rotation modes. The state machine, however, fails to transition between states correctly when moving at different speeds than described in their experiment. Nguyen improved upon this by using Hidden Markov Models and showed that it is possible to predict human behavior in co-manipulation tasks. The algorithm allowed for different speeds of rotation and translation, but ultimately performed worse than Karayiannidis et al.'s method. Neither compared their controller performance to any of the metrics established by other researchers.

Other work has been done by Peternel et al. (2017a) where they incorporated EMG sensor feedback with the control law to provide more information about the stiffness the human was applying in a 1 DoF sawing task. Additionally, Peternel et al. (2017b), in a different work, showed how robots can adapt to human fatigue in pHRI.

One of the few attempts at bi-manual, planar human-robot co-manipulation was developed by Bussy et al. (2012a). Their method relied on force inputs to a trajectory-based control law, where the trajectories are then decomposed into a finite state machine to determine the desired velocities. This research was successful in at least anterior translation coupled with planar rotation, and theoretically generalizes to include lateral translation. However, they do not mention attempts to move in lateral translation, and a video of the controller shows only anterior translation with planar rotation. It is therefore unclear how they deal with the TvR problem.

Others have explored human-robot co-manipulation from the standpoints of roles (Mörtl et al., 2012) to leverage the benefits of precision, strength, and repeatability of a robot and the decision-making and cognitive capabilities of a human. These roles can be allocated, shared, or passed between the human and robot agents to improve performance for different phases of a co-manipulation task. Not surprisingly, researchers found that humans prefer a lower-effort role, offloading more to the robot when appropriate but also taking on more effort at certain times during the task of moving a table on wheels through and around obstacles (Mörtl et al., 2012). Similarly, this continuous adjustment of not just roles but adjustment of control parameters is explored in Sirintuna et al. (2020) and Hamad et al. (2021), in which the researchers study variable admittance controllers as the needs of a collaborative task can change over time. In the later reference, the force is scaled or even amplified to improve the performance of a task (Hamad et al., 2021). These variable implementations of controllers can therefore make trades between, and adjust the emphasis of, transparency and stability of the given system (Aydin et al., 2020). Finally, in Al-Saadi et al. (2023), the authors use a random forest classifier to determine conflict-based interaction states using haptic signals. Their robot then responds appropriately based on a mixture of force-sensing strategies, admittance control, and potential fields to complete a collaborative task.

#### 2.2.2 Motion-based co-manipulation methods

In addition to force-based methods, many insights into human-robot interaction have been gained from studying motion-based intent. One of the common methods of motion-based co-manipulation is using a minimum-jerk basis. Corteville et al. (2007), did so for a 1 DoF point-to-point experiment. Also, Maeda et al. used minimum-jerk trajectories to predict human intent for proactive robot behavior (Maeda et al., 2001). This strategy reduced the amount of effort a human partner needed to exert in co-manipulation tasks, which is one of the problems with variable impedance control.

Interestingly, Thobbi et al. (2011) showed that there are some human movements that are not minimum-jerk movements, but they did not consider higher DoF tasks, nor do they incorporate haptic inputs. Miossec and Kheddar (2008) also explored non-minimum jerk-based trajectories extending the work of Bussy et al. (2012b), where the dyad motions are longer and include walking and not just arm movement.

Ge et al. (2011) showed that machine learning can be a useful tool in pHRI. Their research used supervised learning to predict the motion of the human limb. While their work, along with that shown by Thobbi et al. (2011), shows that human performance can be learned and applied to pHRI controllers, they did not account for co-manipulation of an extended object. Another use of machine learning was demonstrated by Berger et al. (2015) where they used accelerometer and pressure sensor information to learn a statistical model to guide the robot's behavior. However, they did not explore the TvR problem, and it is not clear how well this method performed in comparison to human-human dyads. More recently, Lanini et al. (2018) used a multi-class classifier to determine if a robot should start or stop walking, accelerate, or decelerate for a seemingly one DoF task with a single arm.

### 2.3 Performance metrics

An issue in co-manipulation studies and methods is determining what constitutes a successful dyad. One dyad might take longer than the other, or a dyad might also have more variability in motion than another. Therefore, there needs to be performance metrics that allow for comparison between dyads.

Haptic information has been shown to be a viable communication method, and some researchers have suggested this information is used by dyads to minimize certain criteria. Groten (2011) described a number of these metrics, including minimizing interaction forces and root-mean-square tracking error, and maximizing time on target. A reference trajectory that is commonly used, such as in Corteville et al. (2007) and other previously mentioned studies, is the minimum-jerk trajectory. However, there are also tasks that do not fit well with the minimum-jerk trajectories (Miossec and Kheddar, 2008; Thobbi et al., 2011). Therefore, some alternative trajectories may need to be used if using a root-mean-square error on trajectory.

Ivaldi et al. (2012) also described a few other metrics, such as minimizing jerk, torque change, geodesic trajectories, energy, and effort. These are all fairly well explained by their titles, and the objective of minimizing these metrics is to achieve human-like behavior. More metrics not mentioned by Ivaldi et al., but commonly used in other works are minimizing task completion time (Duchaine and Gosselin's, 2007; Miossec and Kheddar, 2008) and position error in trajectory following tasks such as tracing a path through a maze (Ikeura and Inooka, 1995; Thobbi et al., 2011).

### 2.4 Human intent estimation

One of the main hurdles remaining in human-robot co-manipulation is effective human intent estimation. Many papers have suggested that haptic channels are an appropriate method of communication for human intent (Basdogan et al., 2001; Reed et al., 2007; Groten et al., 2013; Noohi et al., 2016). This makes sense, as we have seen that human teams can move objects by interacting only through forces applied to the objects, rather than by communicating verbally or otherwise (Mojtahedi et al., 2017; Sawers et al., 2017). Many studies have concluded that robots can be controlled by human force input in this manner, but these studies often involve the human acting directly on the robot, and not through any extended object (Ikeura et al., 1997; Rahman et al., 2002; Tsumugiwa et al., 2002; Corteville et al., 2007).

Another method of intent estimation that has been used is programming by demonstration, as in Rozo et al. (2016). Here, intent is compressed into a section of possible motions the human-robot dyad could take. The disadvantage is that it is not robust to disturbances or trajectories that have not been previously modeled. Our definition of intent for co-manipulation of extended objects allows us to capture intent for motion with no definite start or end point (as observed by the robot), or motion that involves unforeseen obstacles.

### 2.5 Related work summary

As has been shown, there are very few studies that look at co-manipulation of extended objects, and even fewer that look at high DoF bi-manual co-manipulation. Approaches for control methods are varied between force-based and motion-based, but almost all are limited in applicability due to low DoF, or lack of generality (requiring previous knowledge about a desired trajectory). We also have not seen a working bi-manual co-manipulation controller for a human-robot dyad, with at least 3 DoF that can be used in undefined situations or respond to disturbances, in any of the related literature.

In our past research, we have completed a human-human dyadic study that required participants to move a large, extended object through many degrees of freedom while recording relevant force and motion data (see Mielke et al., 2017). Based on that data, we compared two different methods for intent prediction, and found that neural networks provided a promising avenue for future efforts (see Townsend et al., 2017). Furthermore, we then developed two data-driven co-manipulation controllers (one based on force inputs, the other on object displacement) that were presented as part of a masters thesis (Mielke, 2018), and pre-print version of this paper (see Mielke et al., 2020). This paper (as opposed to past versions) focuses on the development and comparison of the proposed human-robot co-manipulation controllers.

## 3 Observations and data from human-human experiment

### 3.1 Overview of prior human-human dyadic experiment

**Figure 1:**
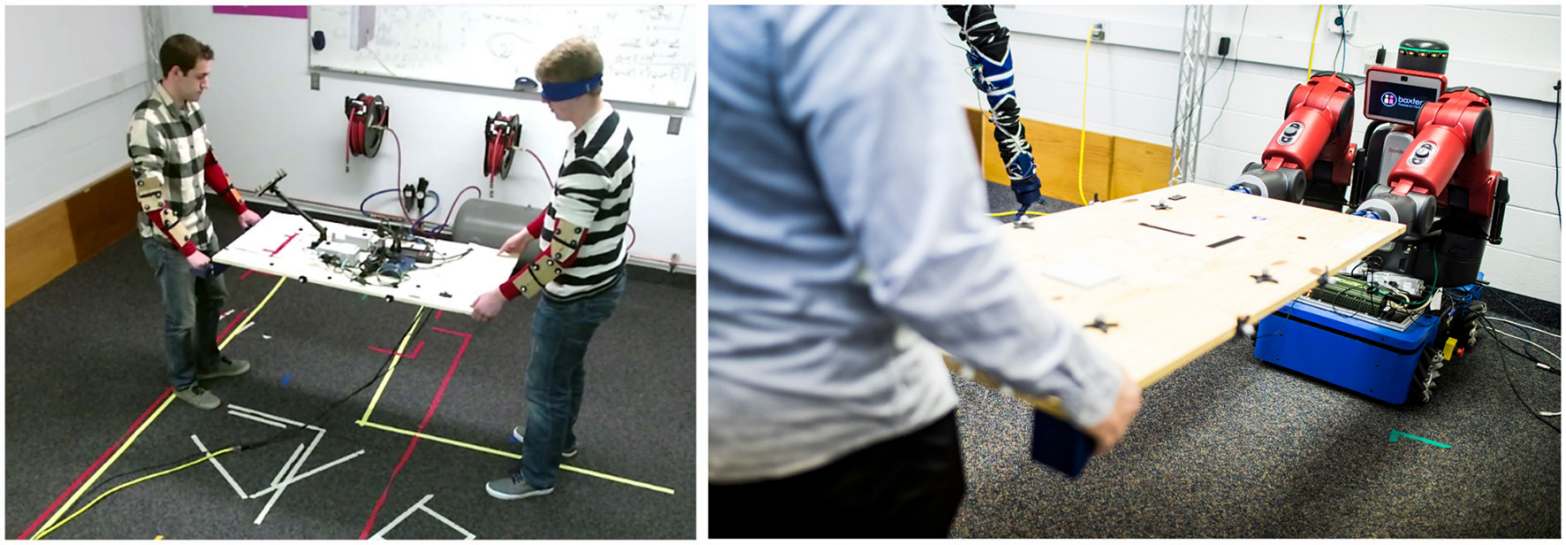
**Left**: A leader and a blindfolded follower performing a table-carrying task. **Right**: Rethink Robotics Baxter robot mounted on a holonomic base carrying the table with a person.

**Figure 2:**
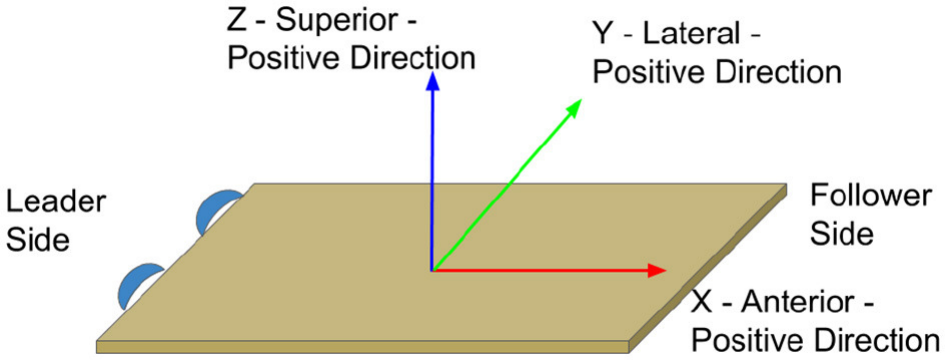
Anatomical direction reference with corresponding table axis: X is anterior, Y is lateral, and Z is superior.

We previously performed a human-human co-manipulation dyad experiment with 21 dyads. Each dyad moved an extended board representing a table or stretcher as we measured their motion and forces on the board as shown in Figure 1. The tasks ranged from one degree of freedom required for the motion of the object, up to, potentially, six degrees of freedom. Each member of the dyad was randomly assigned the role of leader or follower, where the leader was instructed how to complete the task and the follower was expected to assist in completing the task based on communication from the leader. Furthermore, the follower was either blindfolded or not according to a randomized assignment. This was intended to show how people behave when relying solely on haptic feedback, and to give a baseline of performance when human partners are restricted in a communication channel (i.e. vision in this case) while co-manipulating a large or extended object. This study has been both described and analyzed previously in Mielke et al. (2017) and Jensen et al. (2021). In this paper we follow the coordinate frame and sign conventions as described specifically in Mielke et al. (2017) and shown in Figure 2. In this paper, our objective was to use the recorded haptic and motion-based data from the object to enable physical human-robot interaction controllers that would allow intuitive co-manipulation.

### 3.2 Observations relative to in-plane translation vs. rotation

**Figure 3:**
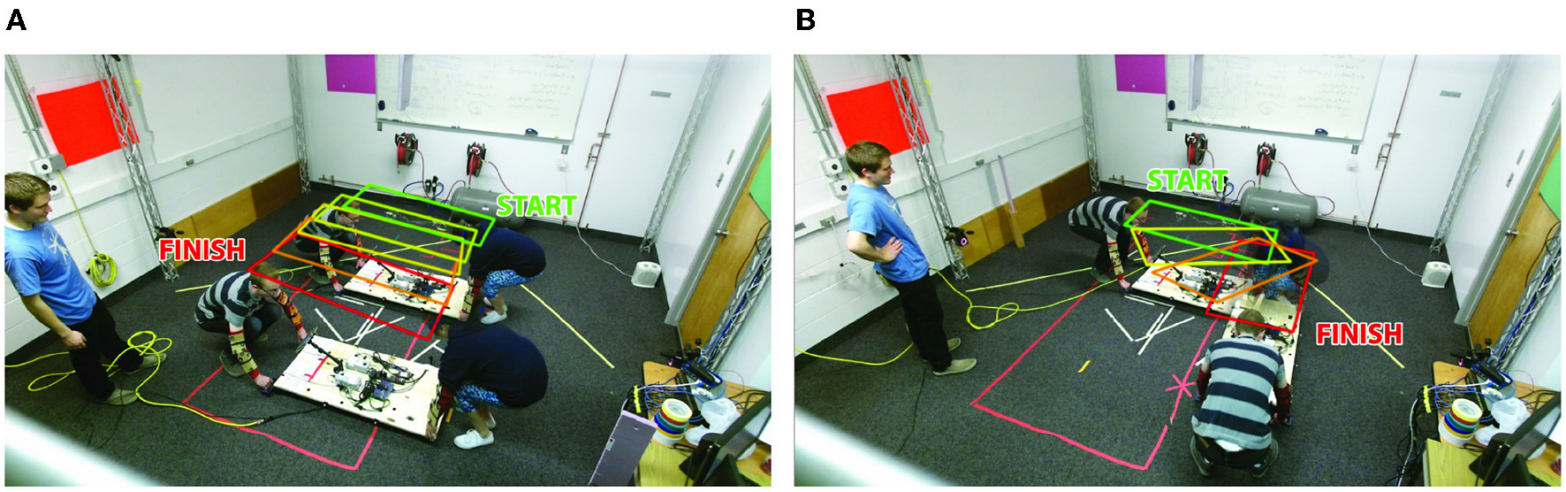
Examples of the simple planar translation and rotation task executed by each H-H dyad and emulated by the human-robot dyads in this paper. Used with permission (Jensen et al., 2021). **(A)** H-H translation task. **(B)** H-H rotation task.

Although the original human-human experiment involved six different tasks with up to six DoF, this paper focuses on determining a control strategy for three DoF planar motion. Since nearly all previous co-manipulation methods involve one or two DoF (mostly for co-located manipulation), three DoF planar motion is a natural step toward our goal of eventual six DoF co-manipulation. Because we are focusing on three DoF planar motion, our observations of the data from the human-human dyad study focus mainly on the blind-folded tasks that required only lateral translation and rotation about the leader or follower (as shown in Figure 3).

The emphasis was placed on these tasks for two main reasons. First, because we perceived a gap in the related literature for three DoF motion of large, extended objects where most past research was focused on co-located co-manipulation, or motion in only the anterior direction. Second, we expect that many more complicated planar motions can be made from combinations of lateral and rotational motion [including other tasks demonstrated by human-human dyads in Mielke et al. (2017)].

In the case of lateral movements (or side-to-side), we recognized some patterns in how the dyads behaved. Studying the videos of the lateral motion task, the follower often guessed the leader's intent incorrectly, and began to rotate when the leader started their movement. When this happened, the leader would exert a force on one side of the table, causing a torque on the table, and the follower would then commence moving in the correct manner. With this video evidence, we looked for in-task patterns of applied torques which could indicate the leader's intent to start either a translation or rotation task.

**Figure 4:**
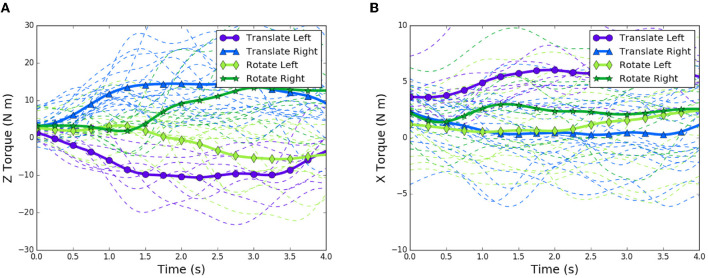
First 4 seconds of trials showing torque trends for rotation and translation tasks for both directions of motion: dashed lines are individual trials, bold lines are averages over all types of trials. **(A)** z-axis torque patterns. **(B)** x-axis torque patterns.

In order to see in-task relationships, we looked at the time series of torque for each relevant task and two distinct groups became obvious. These two groups represented the torque values for the direction of the rotation task, since the dyads were assigned to randomly rotate either clockwise or counterclockwise for each rotation task performed. We then looked at the same z-torque time-series data for the translation tasks, and noticed that two more groups appeared, indicating that there was a difference between translation and rotation tasks, as well as a difference depending on which direction the table was traveling. We took an average of z-torque for each of the 4 distinct groups: translation left, translation right, rotation clockwise (left), and rotation counterclockwise (right). We noticed there appeared 4 groupings of average z-torque for the entire time series. These findings are summarized in Figure 4 and corresponding fixed torque thresholds were identified and subsequently used in the controller described later and represented in Figure 6B.

As can be seen, translation tasks tend to increase in z-torque more quickly, whereas the rotation tasks hover around the same value for over one second before diverging. It is evident from this plot that there is a clear difference in torque patterns between the translation and rotation trials, and also the direction of travel. Based on the z-torque value, the intent can be classified as either translation left, or translation right. However, there is no difference between z-torque patterns for the first second of left and right rotations. This is an important time segment, since it is during this interval that decisions about whether to rotate or translate are made by the follower.

We also identified other signals that might be used to disambiguate lateral translation from rotation from videos of the experiment. We noticed that some dyads tended to rotate the board about the anterior (x) axis while performing the tasks. The results of examining the x-axis torques can be seen in Figure 4. Similar to torques in the z-direction, there is a divide between left translation and right translation. Additionally, a divide appears between left rotation and right rotation. We therefore used the z-torque to determine direction of travel, and x-torque to determine type of motion.

**Figure 5:**
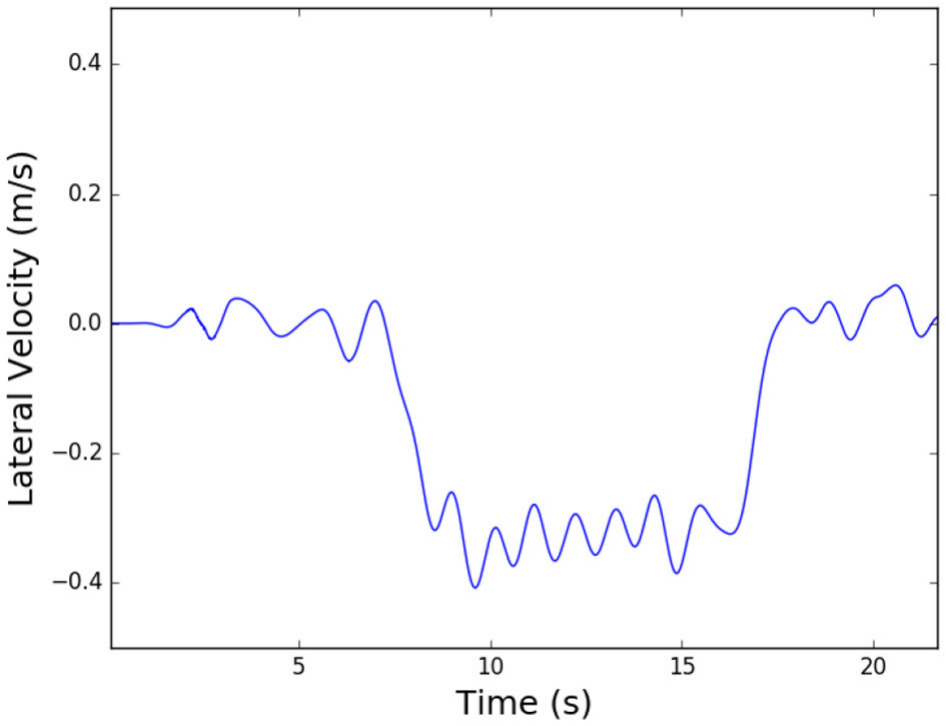
Plot showing lateral velocity profile for the beginning of Task 5, a 3D complex task avoiding obstacles: this portion of the task includes a lateral translation for over two meters.

After determining force-based triggers that would enable a distinction between rotation and translation, we determined what the velocity profile should look like for these tasks if a robot were to act as a teammate. For the translation tasks, we assumed it would follow the bell-shaped velocity profile from a minimum jerk (MJ) trajectory, however, we wanted to first confirm the velocity profile shape when translating over a large distance. Bussy et al. (2012b) showed that humans often accelerate an object to a steady velocity while translating an object. We wanted to verify this, and also determine what velocity most dyads chose as the steady-state velocity. To do this, we looked at our 3D complex task data. This task involved a large translation portion, followed by changes in direction and rotation of the board to avoid obstacles. Figure 5 shows the first portion of a typical complex task, which is a lateral translation for over two meters. We notice from this data that the results seen in Bussy et al. can be verified, and also that the steady velocity achieved is around -0.35 m/s for most dyads. It is important to note that this velocity value is for a 10.3 kg board, and may differ depending on the mass of the object. However, despite this limitation, the observations about torque patterns shown here provide the basis for task disambiguation for a robot follower to use during co-manipulation of extended objects.

## 4 Materials and methods

### 4.1 Robot hardware platform description

Since some of the controller details described in subsequent sections rely on some characteristics of the robot hardware we use, we first describe this hardware.

Our robot platform for this research is a Rethink Robotics Baxter robot mounted on an AMP-I holonomic base from HStar Technologies as seen in Figure 1. There are force/torque sensors on Baxter's wrists, and the base is equipped with mecanum wheels. For our initial work, we chose to use a holonomic base with mecanum wheels instead of a bipedal robot in order to validate that the human intent prediction works at the appropriate speeds without having to incorporate the complexities of bipedal robots. This is important to ensure that our methods work in real-world applications as limiting speed due to limited locomotion may affect the dynamics of the interaction.

For all human-robot experiments described throughout the rest of this paper, the Baxter arms ran an impedance controller with a commanded joint angle calculated for acceptable positioning of the table. The impedance controller was run along with Baxter's built-in gravity compensation. The impedance control law, given in Eq. 1, used *K*_*p*_ and *K*_*d*_ gains of [40, 120, 40, 16, 8, 10, 12] and [7, 8, 4, 7, 1.5, 1.5, 1] respectively. The same gains were used for both arms. The desired angles, *q*_*cmd*_, used were [0, -0.84, -1.27, 2.26, -0.34, -1.22, -2.25] radians and [0, -0.84, 1.27, 2.26, 0.34, -1.22, 2.25] radians for left and right arms respectively. We ran the controller at a rate of 500 Hz.


(1) $$\begin{array}{l} \tau_{cmd}=K_{p}\left(q_{cmd}-q\right)-K_{d}\dot{q} \end{array}$$

As described in other literature, Burdet et al. (2013), the impedance controller allows the robot to react in a more human-like manner, making the human-robot interaction more natural for a human user. While humans typically use their arms in co-manipulation tasks, especially when doing precise placement, using the impedance control law allows us to run initial studies to determine if our co-manipulation controllers are good approximations for human behavior in co-manipulation.

### 4.2 Planar extension of variable impedance control

#### 4.2.1 Motivation and formulation

In order to verify that the torque patterns described in Section 3.2 would be applicable in a human-robot extended object co-manipulation scenario, and also to show that current variable impedance co-manipulation techniques in the literature are not adequate for extended objects, we built an extension for a variable impedance controller. Variable impedance control (VIC) is a possible solution to undefined or indefinite scenarios, since it is not based on a trajectory, but rather on force inputs that determine robot velocity. What we noticed in practice is that VIC causes high internal forces when dealing with bi-manual co-manipulation of an extended object. However, using two arms was essential to being able to carry a heavier, more realistic payload. We first implemented a VIC based on Duchaine and Gosselin's (2007) work, on our robot platform.

**Figure 6:**
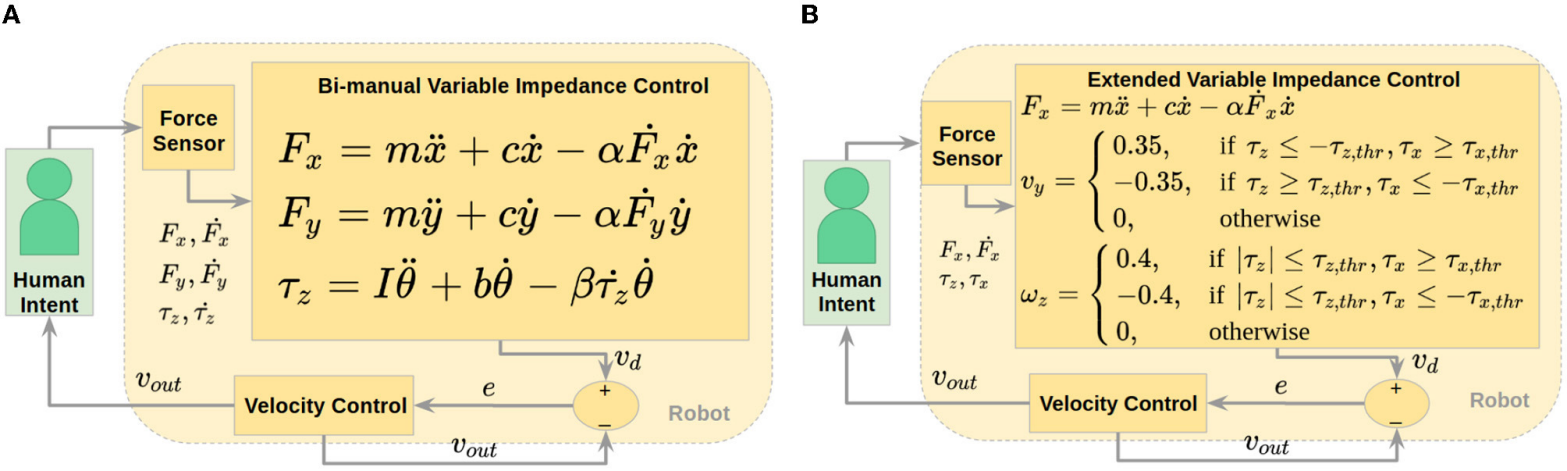
Control loops for co-manipulation of an extended object showing human (in green box) communicating intent haptically through force sensor, then desired velocity is calculated using the specified control law and sent to velocity controller. **(A)** Control loop for BMVIC. **(B)** Control loop for EVIC.

Our implementation of VIC, which we called Bi-Manual VIC (BMVIC), involves the control loop seen in Figure 6A. The human communicates their intent to the robot through force sensors, and the VIC model determines a desired velocity based on the applied force, and how the force is changing in relation to the robot's velocity. The general force model (for x and y directions) is shown in Figure 6A. Here, *F* and Ḟ are applied force and time derivative of force, respectively, ṗ and $\ddot{p}$ are velocity and acceleration, and *m*, *c*, and α serve as virtual mass, damping, and weighting parameters to define the impedance. These virtual parameters do not correspond to the actual parameters of the system. They have values of 1.2, 0.6, and 0.2 respectively, and were determined by trial and error. The model can be discretized and implemented as a discrete LTI system, solving for the desired velocity at each time step. We applied the resulting desired velocity that would give a model impedance directly to the base and controlled the robot arms to have very low impedance (see Section 4.2.2).

This method was developed for single-arm manipulation, so we implemented a VIC for each arm independently in order to achieve bi-manual manipulation. However, this is not an ideal method for bi-manual control. Pushing one arm forward and one arm backward would apply zero net force, causing the robot to remain stationary, rather than rotate as expected. To account for this, we added a torque model to their VIC model, as seen in Figure 6A. Here, τ and $\dot{\tau }$ are applied torque and time derivative of torque, respectively, with $\dot{\theta }$ and $\ddot{\theta }$ as angular velocity and acceleration, while *I*, *b*, and β serve as virtual inertia, damping, and weighting parameters, with values of 0.12, 0.6, and 0.2. All forces and torques referenced here and used for variable impedance control are with respect to the center of the table. The bi-manual torque-based model theoretically allows VIC to be extended to planar motion, where pushing one arm forward and one arm backward will provide a net torque, indicating a desired angular velocity (in the plane only), in addition to any desired Cartesian velocities calculated by the original model. In summary, at each time step, the equations for force and torque are solved to determine desired velocity and angular velocity to send to the velocity controller.

We also extended VIC in a novel way, using our results from Section 3.2. We used the force equation in Figure 6A as a base controller for anterior/posterior desired velocity and added torque-based triggers for lateral translation and planar rotation. The logic of this extended variable impedance control (EVIC) is shown in Figure 5. Torque thresholds are calculated, based on Figure 4, and are implemented as shown. We centered the thresholds around zero for ease of implementation. The threshold values are 3.0 *Nm* for z-torque and 1.5 *Nm* for x-torque. If none of the torque threshold conditions are met, the algorithm commands no lateral translation or rotation about the superior axis. If the torque threshold conditions are met, the robot accelerates until it reaches a specified steady-state velocity. The lateral velocity value, 0.35 m/s, was determined from the logic described in Section 3.2 and Figure 5, and the rotation velocity value, 0.4 rad/s, was determined similarly. The robot acceleration was limited to the capabilities of our robot mobile base. A control loop showing how this algorithm is implemented is shown in Figure 5. The main difference between EVIC and BMVIC is that EVIC uses torque thresholds to determine the desired lateral and angular velocities, whereas BMVIC relies on the equations in Figure 6A to calculate the desired lateral and angular velocities.

#### 4.2.2 Extended object co-manipulation implementation

We implemented both BMVIC, as well as the EVIC on our robot platform, shown in Figure 1. A video showing EVIC running can be seen at https://youtu.be/Vl9kNB0uRLY. Our purpose in implementing both controllers was to determine their feasibility and also to acquire initial data quantifying the performance of a human-robot dyad against the blindfolded human-human dyads. As a reminder, BMVIC is a bi-manual implementation of the most relevant pHRI controller found in related literature (see Section 2) for co-manipulation of an extended object. We ran both BMVIC and EVIC and evaluated them based on the following criteria: lateral translation and planar rotation, or rotation about the superior axis. We ran the controller at a rate of 500 Hz, manipulating or carrying the same table from our human-human dyad experiment (see Figure 1). For determining performance of the controllers, we compared the completion time and MJ error for both lateral and rotational tasks. We also had a qualitative metric: whether BMVIC, EVIC, or neither controller was preferred by the human participants.

##### 4.2.2.1 Pilot study testing

During feasibility testing, we discovered important issues with the BMVIC method. The problem for BMVIC arises when forces are applied laterally on a long object being manipulated by two partners, and the follower does not know whether the leader wants to rotate or translate. We had hoped that introducing an impedance relationship for torque would allow us to overcome the TvR problem. In practice, however, the controller was unable to correctly predict the direction and type of motion desired. Additionally, the robot often moved aggressively with the human in the loop, causing large internal forces in the kinematic chain between the two arms, and shearing internal components within the arm during two different trials. When running EVIC, incorrect predictions occurred, but only when the user did not move as the algorithm anticipated and this movement did not cause aggressive behavior. We recognize this does not allow for a detailed comparison between BMVIC and EVIC. But due to the resulting damage on our robot platforms, we instead decided to only compare EVIC to human-human data from our previous study and to the neural-net-based controller described next in Section 4.3.

### 4.3 Neural network control

**Figure 7:**
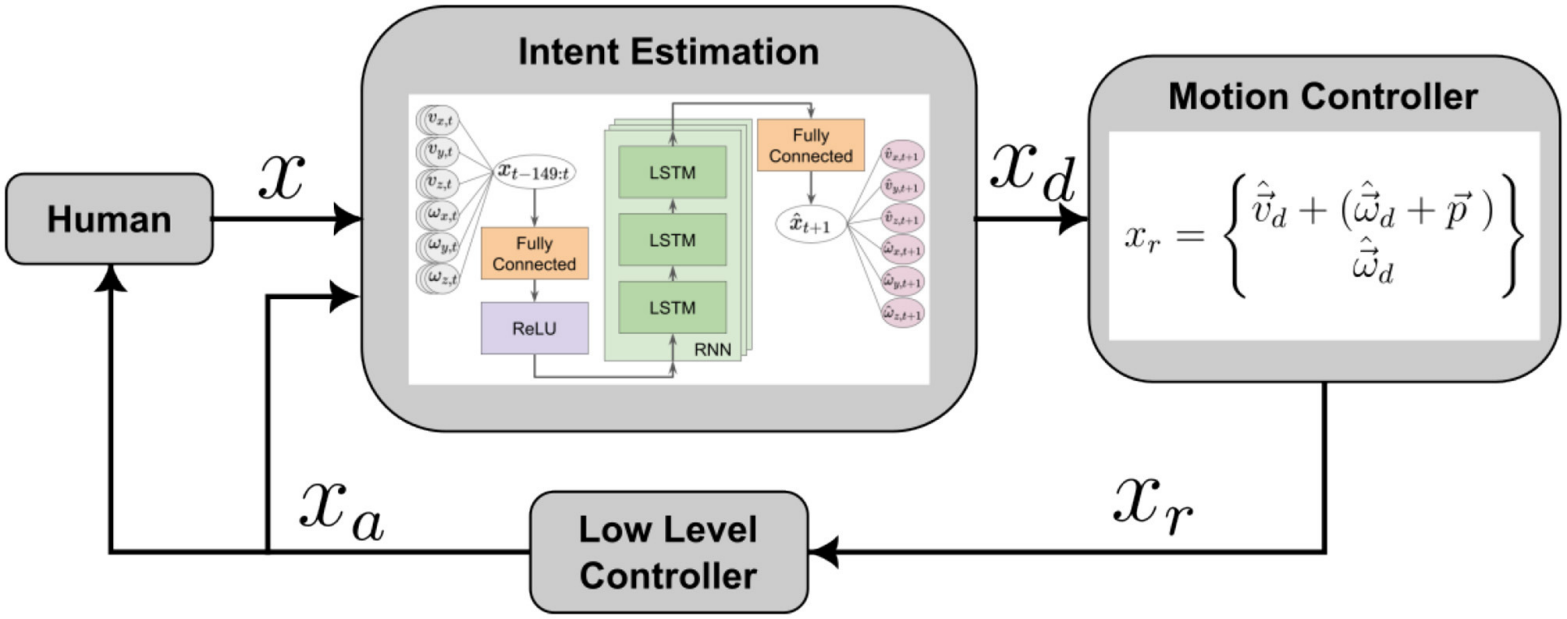
Basic control loop structure of intent estimation in co-manipulation. The human moves the co-manipulated object, and the motion of the object, *x*, is fed into an intent estimator, which determines a desired motion of the robot, *x*_*d*_. The commanded robot's motion, *x*_*r*_, and resulting actual motion *x*_*a*_, then influences the object motion, as well as influencing the human leader. For the network, time-series motion data **(Left)**, which are the inputs, are sent through a fully connected layer, a ReLU layer, an LSTM Cell RNN, and another fully connected layer before predicted velocities are given as outputs **(Right)**.

A more direct approach to intent-based co-manipulation is to estimate the desired motion of the co-manipulated object and have the robot respond accordingly. We, therefore, used Google TensorFlow to develop a neural network that could accurately predict human intent. The output of this intent estimator could be used directly to control the object with a control loop similar to that seen in Figure 7.

Because our data considered the interaction between a human leader and a human follower, the input *x*, could be considered what the leader did–in terms of applying forces or moving the object–to indicate their intent to the follower. The follower then deciphered the intent, *x*_*d*_, and moved as they believed appropriate, *x*_*a*_. Despite the obvious physical interactions between the leader and the follower as they both manipulate the same rigid object, we choose to assume that the signal that we attribute to the leader can be used to directly interpret and predict intent and is what the follower should attempt to respond to.

Among the potential variety of neural network structures that could be considered for this purpose (Sutskever et al., 2011) showed how given a sequence of characters, a Recurrent Neural Networks (RNNs) can be used to predict the next character in the sequence. Leveraging this architecture we had sequences of forces on, and motion of, a table that could be used as inputs to an RNN. We used force and motion data as an analog to characters in other RNNs, and calculate a motion prediction as an output. This prediction encapsulates the human intent, encoded as a desired velocity of the co-manipulated object, and therefore provides a goal for the robot to achieve.

#### 4.3.1 Architecture

We do not explore the effect of multiple different architectures on the performance of our neural network predictor and controller. In addition, although we generated preliminary networks that used both past force and motion to predict future motion, networks that used only motion data (linear and angular velocity of the object) as inputs performed better in our initial trials. We expect that including a dynamic model, changing the RNN structure, or using a different architecture of neural network altogether could allow a better use of force data. However, we have left this for future work given the baseline performance that we were already able to achieve. The structure of the neural network is shown in the “Intent Estimation” block of Figure 7. Our final network consisted of three LSTM layers each with 100 hidden states. Despite the myriad of potential other NN architectures, our purpose in this paper is to show that estimating human intent and incorporating it in a human-robot controller is possible based on the HHI data collected.

Additionally, it was shown by Chipalkatty and Droge (2013) that more complex predictions of future movement can actually decrease performance if they do not agree with what the human is trying to do. They found that it was more important that the human understand what the robot is planning to do, meaning that our controller should be “legible” (see Dragan et al., 2013) for a human partner in a human-robot dyad. In addition to being legible, the prediction should also be accurate and repeatable. The inputs to the neural network, as seen in Figure 7, are 150 past steps of velocity and angular velocity of the table in the x, y, and z directions, {*x*_*t*−149_, *x*_*t*−148_…, *x*_*t*−1_, *x*_*t*_}. The outputs are the predicted velocity and angular velocity of the table in the x, y, and z directions for one time step into the future, ${\hat{x}}_{t+1}$, where $\hat{x}$ indicates a predicted value.

**Figure 8:**
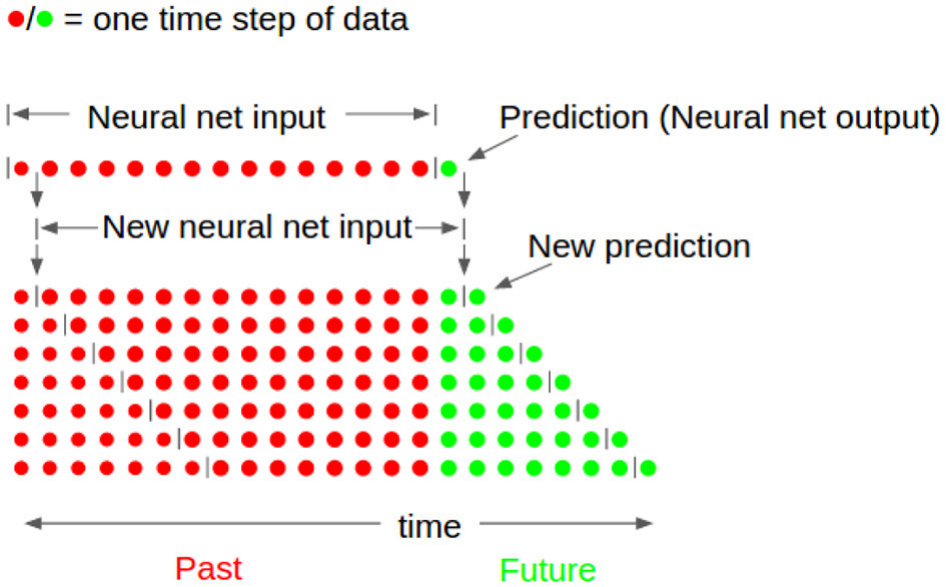
Neural network prediction explanation. Previous time steps (shown in red) are used to obtain one future prediction of states (shown in green). This state is then appended to previous time steps, the first time step is removed, and the network is run again in order to achieve multiple future predictions.

Our neural net formulation also uses what Engel et al. (2004) describe as iterated prediction. The neural network itself only predicts one time step into the future. Then, the prediction, ${\hat{x}}_{t+1}$, is appended to the input to give $\left\{x_{t-149},x_{t-148}...,x_{t-1},x_{t},{\hat{x}}_{t+1}\right\}$. The first step of the input is dropped to obtain a new input of past motions for the neural net, $\left\{x_{t-148},x_{t-147}...,x_{t},{\hat{x}}_{t+1}\right\}$. The new data is input into the neural net which outputs a prediction one step forward, but two total steps into the future, ${\hat{x}}_{t+2}$. This is then appended to the input. The process is repeated 50 times to obtain a prediction of 50 steps, $\left\{{\hat{x}}_{t+1},{\hat{x}}_{t+2}...,{\hat{x}}_{t+49},{\hat{x}}_{t+50}\right\}$. This process is depicted in Figure 8. Because the outputs of each prediction step become the inputs for the next, the inputs and outputs must be the same variables.

#### 4.3.2 Training

We pre-processed the data for the neural net to improve the results. The velocity and acceleration data were scaled to have a mean of zero and a standard deviation of 1 over the entire set of data. This was then inverted on the output to show the results in their proper units. This same scaling can be used on new data as long as the mean and standard deviation are similar to the training data. This is the case in our experiment, as velocity values fall into the average adult human range. The entire set of data consists of 2.5 million time steps for each variable. Data was split into two, training and validation, sets. 75% of the data was assigned to the training set and the other 25% to the validation set.

The neural net was trained in a special way in order to make the iterated prediction ${\hat{x}}_{t+1}$ stable beyond the first step. This process is described in more detail in Engel et al. (2004), and more specifically in Mielke (2018). The neural net predicts 50 steps or 0.25 seconds into the future. This number of steps was chosen because outputs beyond this point did not produce accurate predictions. We speculate that this was due to a limit on the predictability of human intent after a certain amount of time. Humans are inherently unpredictable by nature, and we would not expect that an intent estimator could predict an entire trajectory given only a few data points. Improvements to the neural network architecture may also provide longer prediction times. An additional benefit of this iterated prediction method is that the inclusion of predicted velocities in each training step reduces the amount of overfitting, since new data is essentially being introduced in each iteration.

We trained multiple models for the purpose of cross-validation, making sure that the learned models generalized well across our data set. This included randomly selecting a subset of the data for training and validation for each model to avoid overfitting, similar to k-fold cross-validation.

#### 4.3.3 Validation

**Figure 9:**
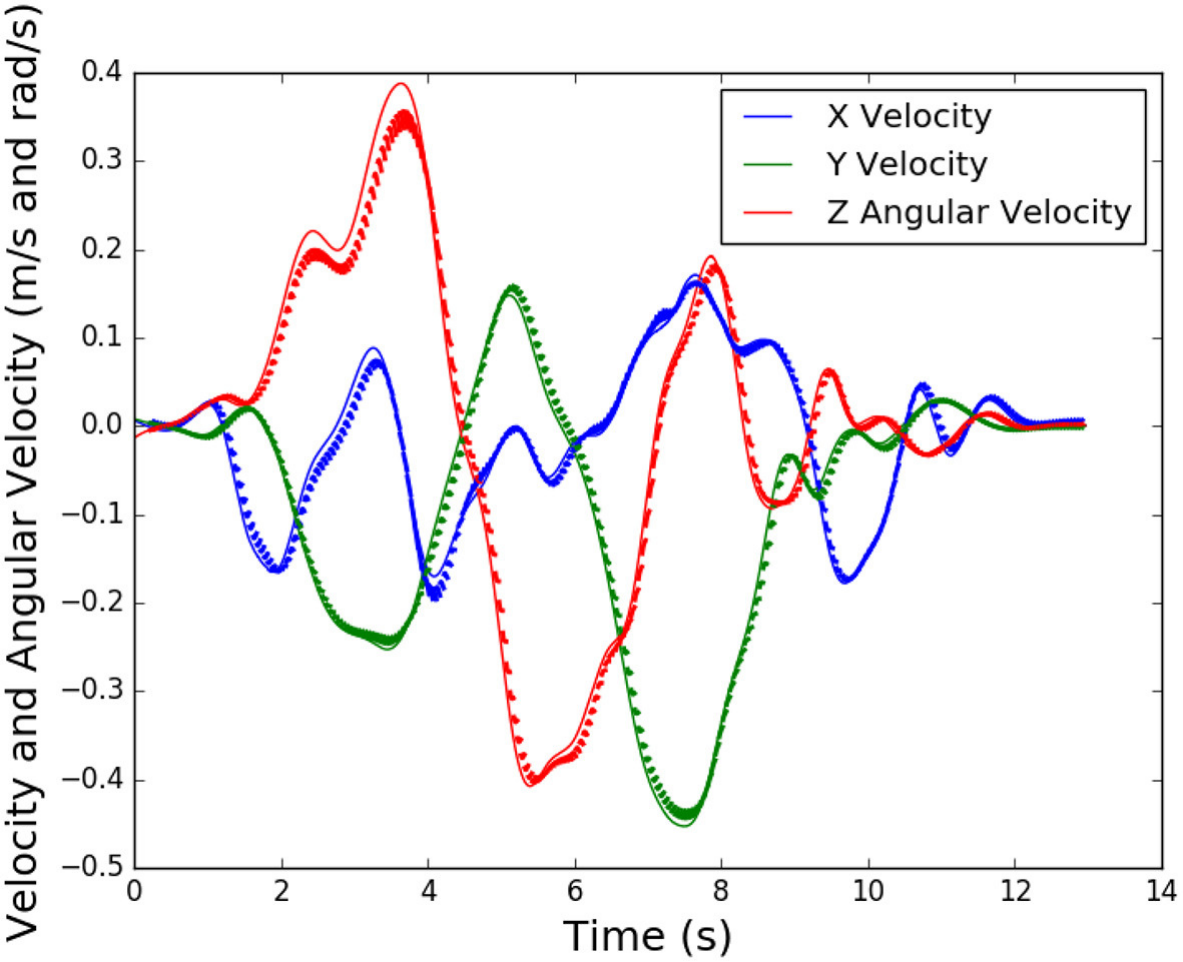
Validation of neural network for a lateral translation task, thin lines are actual velocities and bold lines are predictions for future time steps.

Figure 9 shows the neural network predictions of velocity in the x and y directions, and angular velocity in the z direction for a single sequence of the validation set. The thin lines show the actual velocities, while the bold lines show a 50-time step prediction. These predictions occur at every time step when used for control, but are shown here intermittently (i.e. at 10 second intervals) to improve readability of the plot. As seen, the predictions are reasonably accurate for that time scale. While the prediction deteriorates as we move farther along the iterated prediction, this is acceptable, as only one of the first few predictions will be used for control, and then a new prediction will be generated.

#### 4.3.4 Neural network prediction control

As shown in Figure 7, predicting human intent is only one portion of our proposed approach. We also need to convert the predicted object motion into actual commands for our robot motion controller. The neural network outputs include a predicted velocity and angular velocity of the center of mass (COM) of the table. Given the prediction of the velocity of the COM, we can calculate the velocity of other known points on the table, such as where the robot is gripping the table. However, for our motion controller we shifted the predicted linear and angular velocity of the COM of the object to the COM of the mobile base (assuming they are rigidly connected) to produce a desired velocity for the mobile base. This shifting can be done using the transport theorem shown in Eq. 2. Here, ${\vec{v}}_{r}$ is the robot's calculated velocity in its reference frame, with $\vec{p}$ as the distance from the table frame to the robot frame, and $\vec{\omega }$ as the table's angular velocity in the table frame. Also, ${\vec{v}}_{rel}$ is the table's velocity in its own frame. We assumed the table frame and robot frame do not rotate independently, allowing us to rotate the predicted velocities in the table frame to the robot frame.


(2) $$\begin{array}{l} {\vec{v}}_{r}={\vec{v}}_{rel}+\left(\vec{\omega }\times \vec{p}\right) \end{array}$$

We now have the components to complete the control loop shown in Figure 7. The intent estimator consists of the neural network model. The motion controller is described by Eq. 2, and is subsequently fed into the low-level control of the robot's mobile base, which sends voltages down to the wheels to match the desired velocity. The achieved velocity, *x*_*a*_, is then what the human interacts with, completing the loop. *x*_*a*_ is estimated using numerical differentiation and a 2nd-order low-pass filter of the pose information coming from the motion capture. This loop is shown in Figure 7. We call this control method Neural Network Prediction Control (NNPC). A notable feature of this method is that the commanded velocity, *x*_*r*_, is a continuous variable on [−*v*_*max*_, *v*_*max*_], where *v*_*max*_ is determined empirically for each DoF based on HHI data. This means the human user has control of the speed of the interaction, so if the response *x*_*a*_ is not suitable for the human, they can adjust their inputs to move faster or slower.

### 4.4 PHRI co-manipulation study

As mentioned in Section 4.2, EVIC works only for 3 DoF planar control–anterior and lateral translation and rotation in the plane–so we developed an experiment to compare a planar implementation of NNPC and EVIC. We believe that since NNPC can provide predictions for all 6 DoF, it can be expanded to control in 6 DoF. However, we have left that for future work as it would also require integration with better robot arm control and is beyond the scope of this paper. This experiment was designed to be as close as possible to the lateral translation and planar rotation tasks from HHI data in Mielke et al. (2017).

#### 4.4.1 Experiment description

##### 4.4.1.1 Translation vs. rotation tasks

**Figure 10:**
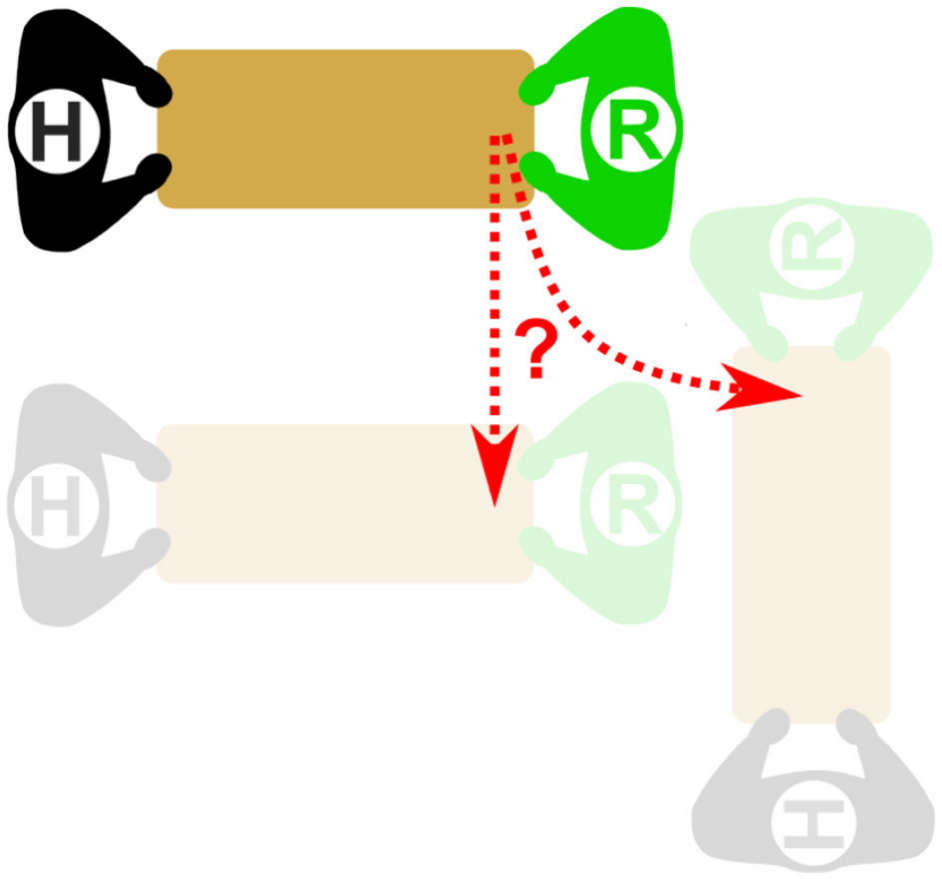
Representation of the ambiguity of a translation task (moving from the top to bottom left) and a rotation task (rotating from the top to bottom right), where Agent R represents a robot, and Agent H represents a human. Agent R will, at least initially, “sense” the same signal or force due to the extent of the object immediately after Agent H initiates movement to either of the final positions.

Figure 10 shows a representative diagram of the tasks to be performed by each human-robot dyad, and the inherent uncertainty in determining which of the two main motions is being attempted. Each participant performed two tasks: translation and rotation. In this diagram, the human is represented by the agent with an “H” and the robot is represented by an “R”. The translation task consisted of the subject moving laterally, either right or left, with tape lines extending on the ground to help the user align the board correctly. Rotation tasks were similar, except with the participant rotating ±90 degrees relative to their starting location. Tasks could be run starting in either configuration, and the direction was randomized throughout the trial. An example of the expected motion during an actual trial can be seen at https://youtu.be/QQKpT1ORxkw.

##### 4.4.1.2 Equipment

The position of the board was tracked via Cortex Motion Capture software with a Motion Analysis Kestrel Digital Realtime System. A total of eight Kestrel cameras were used to track eight infrared markers placed on the object. Using a static global frame established by the motion capture system, the position and orientation of the board could be tracked over time, and we transformed the data into the robot's frame for use in the neural network. The motion capture data was collected at a rate of 200 Hz. In order to run NNPC, we need a method of estimating the object's velocity. We used a 2nd-order, low-pass filter and numerical differentiation on position and orientation data to define the object velocity. Additionally, participants wore sleeves with infrared markers to track the position of their arms during the experiments. This data was not used during analysis but was collected to match similar data collected during the experiment in Mielke et al. (2017).

The object the teams moved was a 59x122x2 cm wooden board weighing 10.3 kg—meant to simulate an object (like a table or stretcher) that is difficult for one person to maneuver. Attached to the board on the side of the robot was a pair of ABS 3D-printed handles, to which two ATI Mini45 force/torque sensors were fastened. The sensors transmitted data via ATI NET F/T Boxes, which passed data over Ethernet to the computer at a rate of 100 Hz. The sensor is attached to wrist adapters on the other side, which fasten to Baxter's wrists.

The test arena was a volume measuring 4.0 × 5.1 × 2.5 m. The arena was also equipped with a video-capturing device. The device used was a Microsoft Kinect 2, which allowed us to capture 3D point cloud data, as well as color video of each trial. Although we did not use the point cloud data for analysis in this paper, the data may be useful in future work.

##### 4.4.1.3 Subjects and procedure

Subjects for this study were male and female students from Brigham Young University in Provo, UT. There were a total of 16 students–4 female and 12 male–ranging from 18 to 23 years of age, with an average age of 20. Students were from a variety of majors, with STEM majors making up a majority. Participants were asked to rate their familiarity with robots on a scale from 1 to 5, with 5 being the most familiar and 1 being not familiar at all, with the average rating calculated at 2. IRB approval was obtained for this experimental study.

Participants entered the Robotics and Dynamics Lab, and provided written informed consent in accordance with IRB. They were then briefed on the purpose of the research and given an introduction to what data would be collected, and what would be expected of them. Sleeves were then placed on the participants' arms in order to track their arm motion during the trial. Subjects were then given basic operating instructions for both EVIC and NNPC controllers. This instruction included how to translate in the anterior and lateral directions, and how to rotate the board for each controller. A controller was randomly selected, and each participant practiced with that controller until they were able to complete a competency task, they moved on to the other controller, and repeated the competency training. The competency task consisted of aligning the board with the tape lines on the ground, starting from a translated and rotated position. The practice assured us that each participant would have at least enough familiarity to complete the translation and rotation tasks.

Once competency training was completed, a controller was selected at random to be the first controller for data collection. The randomization of controllers was counterbalanced. Participants knew the controllers only as option “A” (NNPC) or “B” (EVIC). They were not given any specific details about the formulation of the controllers, other than the basic operating instructions in the competency task. The subjects then ran a series of translation and rotation tasks with the selected controller. Tasks were randomized (counterbalanced) in order between translation and rotation. Once a type of task, either rotation or translation, was selected, the participant ran that task type in one direction (i.e. to the left or to the right), and then ran the same task type, but in the other direction. Due to the nature of the controller, the robot was not able to lift the table from the ground, so the table was laid on a rest stand between trials. A single trial consisted of the subject lifting the table from the stand, then a researcher would remove the stand from below the table. Once the rest stand was completely out of the way, the subject then performed the specified task. Participants indicated they were finished by verbally communicating completion. Once they indicated they had completed the task, a researcher would replace the rest underneath the table, and the participant would lower the table back onto the rest. Each task was repeated six times, three in one direction and three in the other direction, for each controller. Once trials were completed for one controller, the participants were given a survey, and asked to rate the first controller on certain qualitative characteristics. Once completed, they moved on to the other controller.

A video showing the performance of both controllers (EVIC and NNPC) can be seen online at https://youtu.be/Vln9x0CaMXg. This video was taken after the participant had completed all trials, and is a representation of the skill level of the human-robot dyad post-experiment.

## 5 Results and discussion

### 5.1 Evaluation metrics

A number of metrics could be used to quantify the performance of the controllers and a high-level summary of these potential metrics is found in Ivaldi et al. (2012). Among these are a few that are especially applicable to the tasks and control methods developed in this paper including minimum jerk, minimum torque change, and completion time. While none of these metrics can individually store all the information of each controller, collectively they provide a reasonable indication of how each controller performs in relation to HHI data from Mielke et al. (2017).

Minimum jerk error (MJE), or deviation from a minimum-jerk trajectory, is a measure of how close the actual trajectory was to a minimum-jerk trajectory in meters (for translation) or radians (for rotation), is calculated using Eq. 3, and accounts for a human's tendency to match these trajectories. Completion time is the time from the start of the task to the end of the task. We define “start” and “end” as being when the object has moved 5% beyond the initial position (or within 5% of the final position) relative to the *y* positions (or θ_*z*_ for rotation) respectively. A buffer of 0.5 s is added to the total time to approximately account for the missed motion and to give an accurate measure of actual time requiring movement.

Minimum-torque measure (MTM) computes how much the time-derivative of torque changes over the course of the task. In instances where the follower predicted incorrectly, there was an unforeseen obstacle, or some other disturbance, MTM can account for a human's tendency to reduce the amount of force or torque required to move an object, with MTM calculated using Eq. 4.


(3) $$\begin{array}{l} MJE=\overset{T}{\underset{t=0}{\sum }}|x_{mj,t}-x_{a,t}| \end{array}$$

(4) $$\begin{array}{l} MTM=\overset{T-1}{\underset{t=0}{\sum }}{\dot{\tau }}_{t}^{2}+{\dot{\tau }}_{t+1}^{2} \end{array}$$

### 5.2 Quantitative results

While each task type was performed six times for each controller, we only consider the data from the last two trials performed since participants would learn throughout the experiment with the final trials most representative of the particular controller. This assumption is justified as real-world human-robot teams would almost always include some training and familiarization with the robot before deployment.

Using the metrics previously defined, Table 1 compares the EVIC and NNPC controllers, as well as the lower (blind-folded HHI) and upper (sighted or non-blind-folded HHI) bounds of human performance. Overall, NNPC performed the best in most of the metrics. NNPC approached the blind-folded HHI performance in completion time (i.e., 7.75 s vs. 7.18 s for Translation). NNPC also outperformed EVIC, blind-folded, and sighted HHI performance in both MJE and MTM (where lower numbers for a given row in Table 1 indicate more efficient performance in that task). EVIC, while not quite as good, still outperformed blind-folded and sighted HHI in most of the metrics, except for completion time. It is notable that the blind-folded HHI performance captured here is for a human-human leader-follower dyad, where the follower was blindfolded, and communication was limited to haptic communication only, whereas sighted HHI allowed for communication in any form desired by the dyad.

**Table 1:** Performance metrics of EVIC and NNPC for rotation and translation tasks, compared against blindfolded HHI and sighted HHI data from Mielke et al. (2017).

**Task type**	**Metric**	**Unit**	**EVIC**	**NNPC**	**Blind-folded HHI**	**Sighted HHI**
Rotation	Completion time	s	8.25	8.26	7.08	6.58
Translation	Completion time	s	7.91	7.75	7.18	4.93
Rotation	MJE	rad	96.44	87.38	392.71	344.70
Translation	MJE	m	50.24	48.51	149.91	98.92
Rotation	MTM	N^2^·m^2^/s^2^	65,603	12,771	4,88,454	3,41,253
Translation	MTM	N^2^·m^2^/s^2^	48,192	15,221	3,87,938	1,51,759

For statistical analysis, we ran an unpaired *t*-test and determined Cohen's d-effect size for the various factors, controllers, and metrics described above, to ascertain the difference between treatments and the strength of those comparisons. Effect sizes were calculated, and then categorized into very small, small, medium, large, very large, or huge categories, based on Sawilowsky (2009). The statistical results are summarized in Table 2.

**Table 2:** Statistical significance and comparisons of quantitative performance metrics.

	***p*** **-value**			**Cohen's d effect size**		
**Comparison groups**	**Comp. time**	**MJE**	**MTM**	**Comp. time**	**MJE**	**MTM**
EVIC vs. NNPC Trans.	0.73	0.89	0.017	Small	Medium	Large
EVIC vs. NNPC Rot.	0.98	0.70	0.14	Very small	Small	Medium
EVIC vs. Blind-folded Trans.	0.07	0.00	0.00	Medium	Huge	Huge
EVIC vs. Blind-folded Rot.	0.06	0.00	0.00	Medium	Very large	Very large
NNPC vs. Blind-folded Trans.	0.16	0.00	0.00	Medium	Huge	Huge
NNPC vs. Blind-folded Rot.	0.05	0.00	0.00	Medium	Very large	Very large
EVIC vs. Sighted Trans.	0.00	0.00	0.00	Huge	Large	Very large
EVIC vs. Sighted Rot.	0.01	0.00	0.00	Large	Large	Large
NNPC vs. Sighted Trans.	0.00	0.00	0.00	Huge	Large	Huge
NNPC vs. Sighted Rot.	0.01	0.00	0.00	Large	Large	Very large

A few key results are important to recognize from this analysis. First, EVIC and NNPC are not statistically different in terms of completion time or MJE. They do seem to differ in MTM, which has a fairly large effect size. Second, both EVIC and NNPC are not statistically different from the blind-folded human-human dyads in terms of completion time.

Lastly, EVIC and NNPC are statistically different from both blind-folded and sighted human-human dyads in terms of minimum-jerk error and MTM, and these comparisons are all categorized as large or higher. Overall, the statistics show that these controllers have approached a level comparable to blind-folded human-human dyads with respect to the completion time metric, but are sometimes orders of magnitude better than human-human dyads in terms of MJE and MTM metrics. Although we have defined MJE and MTE metrics with lower values as being more desirable, it is interesting to note human dyads may not in fact be minimizing these values. This result may require re-thinking the utility of these metrics in the context of this type of extended object with associated geometry and mass, especially in cases where mimicking human behavior is a desired attribute of human-robot dyads.

**Figure 11:**
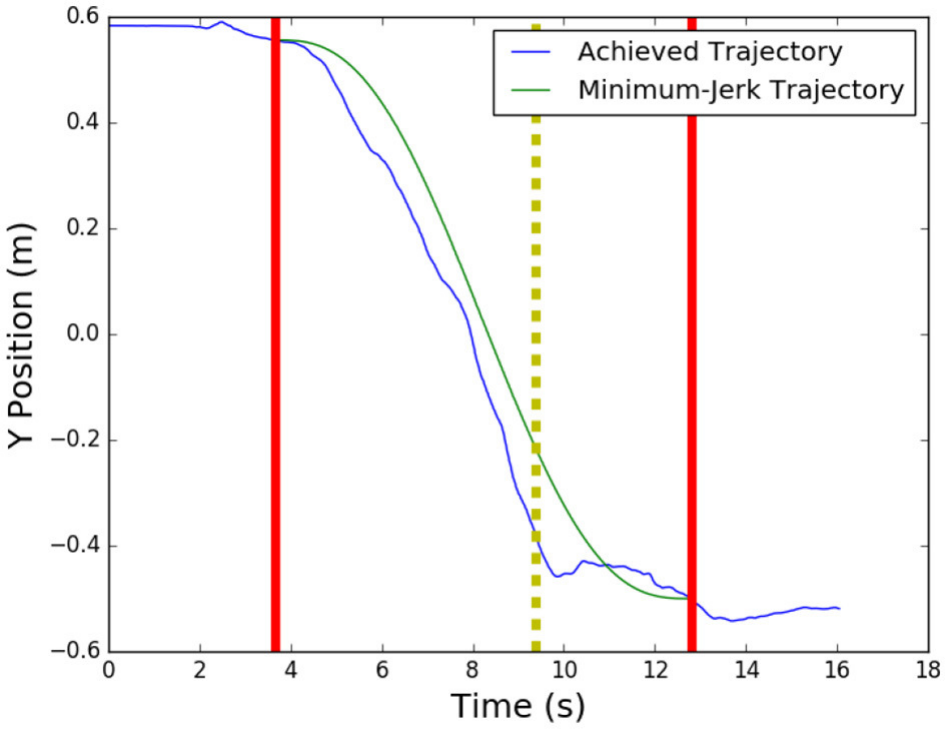
Undershooting behavior of a human-robot dyad for a translation task, where bold, vertical lines indicate start and stop points, and dashed vertical line indicates the 90% completion point. Movement after this point is considered a fine motor adjustment.

Another noteworthy observation is that both EVIC and NNPC, while capable, have difficulties with fine-motor adjustments. Throughout the trials, participants occasionally overshot or undershot their desired position, and had to make fine motor adjustments to achieve the desired final position. An example of undershooting is shown in Figure 11. The dyad is able to complete 90% of the task, represented by the dashed vertical line, in just under 6 seconds, but spends approximately 3 seconds trying to complete the remaining 10%, which amounts to about 10 cm of movement, with more fine adjustments.

On average, the remaining time at 90% completion was 2.40s for EVIC and 2.55s for NNPC. From this data, it would appear that EVIC is slightly better at fine-motor adjustments than NNPC, since EVIC had a smaller discrepancy between achieved and minimum-jerk 90% completion time.

To determine if a few underperforming dyads skewed the average, we also took the median 90% completion time. For achieved and minimum-jerk trajectories, respectively, with EVIC, this gave values of 1.98 and 2.11 s. Similarly for NNPC, it gave values of 1.97 and 2.02 s. From these results, we conclude that this data is positive skewed, and only a small number of dyads had trouble with fine-motor adjustments, causing the higher mean values. Therefore, we can conclude the lack of fine motor skills in the controllers did not significantly hamper their ability to complete the tasks, but should be addressed in future work to help improve the performance of those dyads who struggled with undershooting or overshooting.

### 5.3 Qualitative results

After each participant had performed all tasks with one controller they were asked questions regarding how they thought their partner, in this case, a robot, performed the actions. This survey was repeated after tasks with the second controller. Using a 5-point Likert scale, 1–Strongly Disagree–to 5–Strongly Agree, participants answered 12 questions rating their partner's helpfulness, predictability, speed, and other attributes. The full set of questions can be found in Mielke (2018). Although these questions were not asked in multiple ways (such as in Kucukyilmaz et al., 2013 to detect inconsistencies) the results are useful to assess qualitatively the perceived attributes of the two methods from the perspective of the participants.

The average for each controller rating is given in the first 2 columns of Table 3 with the controller that performed better in each category designated in bold text. For some categories, like *Too Slow*, a lower number is desired, whereas for others, like *Safe*, a higher number is desired. For comparisons, the same survey questions, except for the *Correct Direction* question, was given to the human dyads after the HHI study, with the results shown in the third column of Table 3. Only the responses of the human designated as the leader from the human-human dyads are included akin to the one human in the human-robot dyad experiments.

**Table 3:** Ratings and statistical significance of survey questions, with 5 as strongly agree and 1 as strongly disagree.

	**Controller**		**EVIC vs. NNPC**		**HHI survey**
**Attribute**	**EVIC**	**NNPC**	*p*	**Cohen's d effect size**	**Leader only**
Helpful	3.88	3.88	0.5	Very small	4.52
Fast enough	3.38	3.63	0.19	Medium	4.38
Too slow (^*^)	3.31	2.94	0.15	Medium	2.09
Confusing (^*^)	3.38	3.5	0.34	Small	2.09
Correct task	3.94	3.75	0.28	Medium	4.42
Safe	4.5	4.44	0.36	Small	4.71
Correct speed	3.44	3.56	0.37	Small	4.47
Correct direction	3.5	3.56	0.41	Small	N/A
Good force amount	3.44	2.81	0.04	Medium	4.47
Predictable	3.63	3.5	0.35	Small	4.28
Better than alone	3.5	3.56	0.38	Small	4.38
Equal share	3.75	3.5	0.15	Medium	4.23

Bold numbers indicate a preference between EVIC and NNPC for the specified attribute. Starred (^*^) attributes indicated a desire to minimize values.

For each question, we ran an unpaired t-test to calculate a p-value and determined the Cohen's d effect size presented in Table 3. Only the *Good Force Amount* question obtained a *p*-value of < 0.05, suggesting it is statistically significant. However, this question, as well as a number of others, had a medium effect size.

### 5.4 Discussion

From Table 3, people still clearly prefer working with a human partner over a robot partner as evidenced by the higher values in the last column to either of the controllers. One reason for this may be that humans do not trust robots entirely, as is evidenced by the 5th, 7th, and 8th questions in the survey, which all ask about trust in the partner. Perhaps the same pHRI experiment, with a blindfold and earmuffs on the human would have returned more favorable ratings for the robot controllers.

As was mentioned above, NNPC was the more capable controller in terms of performance metrics. This corresponds to the slightly higher average scores for NNPC compared to EVIC for metrics related to performance *Fast Enough, Too Slow, Correct Speed, Correct Direction*, and *Better than Alone*, but those results were not statistically significant (see Table 3).

Although NNPC users experienced less force overall, based on the TMT metric, the survey indicated that EVIC applied more appropriate forces. From these observations, we surmise that haptic communication is a large factor in how humans perform co-manipulation tasks successfully. Furthermore, EVIC and NNPC were only statistically different in terms of the MTM metric and the *Good Force Amount* question. From these results, we can conclude that NNPC is not applying sufficient or appropriate forces, and is therefore considered more difficult and less intuitive to use by the participants. These results agree with Chipalkatty and Droge (2013), who indicated that training a controller to be the most efficient or best-performing controller may cause it to be a less preferable controller to humans. So while NNPC may potentially be the better-performing controller, EVIC might currently be a more intuitive and appropriate controller for real-world applications with humans, since it applies more appropriate forces.

In terms of completion time data presented in Table 2, we see that both EVIC and NNPC are not statistically distinguishable from the blind-folded human-human dyads. While this is an encouraging result, we know that there is some missing information in our model. Although similar in completion time, our controllers performed much differently than human-human teams in the TMT and MJE metrics. These considerations should be explored further in future controller development for pHRI.

In addition, the EVIC and NNPC algorithms represent the average behavior of 21 human-human dyads manipulating a specific object. It is not evident that the thresholds or learned neural networks would work for objects of different size or mass. However, in our initial testing of the controller, we used a table of about half the length and mass of the table used in the experiment, and achieved similar general performance of the controllers. This generalized behavior, however, was not tested thoroughly.

For EVIC in particular, in order to set thresholds for torque, as well as the target velocity, one may consider using a learned approach–or an optimization–where a user would manipulate the object for a certain period of time, and the algorithm would adjust to the preferences of the user and the characteristics of the object, based on the applied forces and achieved velocities. Similar on-line strategies could be applied to learning the desired trajectory behavior from an individual human partner as part of NNPC.

In terms of limitations of the methods presented in this paper, one of the main issues is knowing whether or not the thresholds found for EVIC, and the models learned for the neural network-based controller, would generalize to other objects with varying mass and geometry. The velocity data for the neural network was normalized. So, given additional training data for a new object we would expect the same approach to work. However, making a learned co-manipulation model more general is desirable in future work. Specifically, we would start by testing if both the neural network model and EVIC thresholds generalize to other objects (since it is possible that they do). However, if they did not, we would expect that scaling the thresholds for EVIC based on the mass and extent of the object would be a reasonable first approach that could be readily validated. While for the neural network, training with multiple objects, and including object-related information in the net would likely help the model to generalize to objects on which the network was not trained.

Finally, one additional limitation is that it is not clear that the force/torque patterns seen in these tasks, nor the torque thresholds used, would be applicable to tasks involving higher DoF. However, because the NNPC was trained on six DoF data, we expect that it may generalize more easily if implemented using the additional degrees of freedom available from the robot arms. This is something that must be explored in future work as we extend our methods to six DoF tasks.

## 6 Conclusion

In this paper, we have discussed the problems and limitations of many current co-manipulation pHRI controllers, especially as they relate to co-manipulation of extended objects in the ambiguous situation of translation versus rotation tasks. We described the key takeaways from HHI experiments gathering the force and motion data for tasks that could inform how humans disambiguate translation versus rotation in the plane. We then apply this data to the development of control methods to enable human-robot dyads to adapt to this ambiguous situation.

Developed from this data, our implementation of an Extended Variable Impedance Control (EVIC), a novel method for planar 3 DoF co-manipulation of extended objects, has certain advantages over standard Variable Impedance Control, as well as Bi-Manual Variable Impedance Control, an extension of a controller from related work. Furthermore, we have shown that human intent can be estimated accurately from the previous motion of the object that is being co-manipulated and that an RNN (coupled with basic motion controller to make a NNPC) with velocity inputs is capable of capturing human intent in the form of velocity estimation.

We found that NNPC outperformed EVIC in all metrics considered and that both were comparable to blind-folded human-human dyads in terms of completion time. Although NNPC was the superior controller based on performance, participants preferred EVIC, claiming they felt it was safer, less confusing, and more predictable (although not at high enough levels to establish significance). We conclude that NNPC sacrifices some intuition for performance, but since the added performance capabilities are unfamiliar to human partners, future users may feel less comfortable than with the force-based EVIC.

## Data availability statement

The raw data supporting the conclusions of this article will be made available by the authors, without undue reservation.

## Ethics statement

The studies involving humans were approved by Brigham Young University Institutional Review Board. The studies were conducted in accordance with the local legislation and institutional requirements. The participants provided their written informed consent to participate in this study. Written informed consent was obtained from the individual(s) for the publication of any potentially identifiable images or data included in this article.

## Author contributions

EM: Conceptualization, Data curation, Formal analysis, Methodology, Software, Validation, Visualization, Writing—original draft, Writing—review & editing. ET: Data curation, Methodology, Writing—original draft. DW: Conceptualization, Methodology, Software, Supervision, Writing—review & editing. JS: Formal analysis, Visualization, Writing—original draft, Writing—review & editing. MK: Conceptualization, Formal analysis, Funding acquisition, Methodology, Project administration, Resources, Supervision, Writing—original draft, Writing—review & editing.
